# Ecological and geographical speciation in *Luciliabufonivora*: The evolution of amphibian obligate parasitism

**DOI:** 10.1016/j.ijppaw.2019.09.005

**Published:** 2019-09-22

**Authors:** G. Arias-Robledo, R. Wall, K. Szpila, D. Shpeley, T. Whitworth, T. Stark, R.A. King, J.R. Stevens

**Affiliations:** a Biosciences, College of Life and Environmental Sciences, University of Exeter, UK; b School of Biological Sciences, University of Bristol, UK; c Department of Ecology and Biogeography, Faculty of Biology and Environmental Protection, Nicolaus Copernicus University, Toruń, Poland; d E.H. Strickland Entomological Museum, Department of Biological Sciences, University of Alberta, Canada; e Department of Entomology, Washington State University, Pullman, USA; f Reptile, Amphibian and Fish Conservation Netherlands (RAVON), Nijmegen, the Netherlands

**Keywords:** Obligate parasitism, Amphibian parasitism, Myiasis, *Lucilia*, Host specialisation, Blowfly

## Abstract

*Lucilia* (Diptera: Calliphoridae) is a genus of blowflies comprised largely of saprophagous and facultative parasites of livestock. *Lucilia bufonivora*, however, exhibits a unique form of obligate parasitism of amphibians, typically affecting wild hosts. The evolutionary route by which amphibian myiasis arose, however, is not well understood due to the low phylogenetic resolution in existing nuclear DNA phylogenies. Furthermore, the timing of when specificity for amphibian hosts arose in *L. bufonivora* is also unknown. In addition, this species was recently reported for the first time in North America (Canada) and, to date, no molecular studies have analysed the evolutionary relationships between individuals from Eastern and Western hemispheres. To provide broader insights into the evolution of the amphibian parasitic life history trait and to estimate when the trait first arose, a time-scaled phylogeny was inferred from a concatenated data set comprising mtDNA, nDNA and non-coding rDNA (*COX1*, *per* and *ITS2* respectively). Specimens from Canada, the UK, Poland, Switzerland, the Netherlands and Germany were analysed, as well as individuals from its sister taxa, the saprophage *Lucilia silvarum* and a Nearctic species also implicated in amphibian myiasis, *Lucilia elongata*. Obligate amphibian parasitism appears to have arisen ~4 mya, likely as a result of niche displacement of a saprophagous/facultative parasite ancestor. Consistent paraphyly of *L. bufonivora* with respect to *L. elongata* across single-gene phylogenies and high mtDNA genetic distances between Nearctic and Palearctic individuals suggest on-going cryptic speciation facilitated by geographical isolation. These findings suggest that recent reports of *L. bufonivora* in the Nearctic do not constitute a recent introduction, but instead suggest that it remained unrecorded due to taxonomic confusion and low abundance. This is the first study to confirm the involvement of *L. bufonivora* in amphibian myiasis in Canada using DNA-based identification methods.

## Highlights

• Within *Lucilia*, a small genus of blowflies mostly comprised of carrion-breeding species, obligate parasitism for amphibians evolved around 4 mya.
• Geographic isolation between Nearctic and Palearctic lineages is facilitating on-going cryptic speciation of *Lucilia bufonivora*.
• First positive identification of *L. bufonivora* from two confirmed cases of amphibian myiasis in North America.

## Graphical abstract

**Figure:**
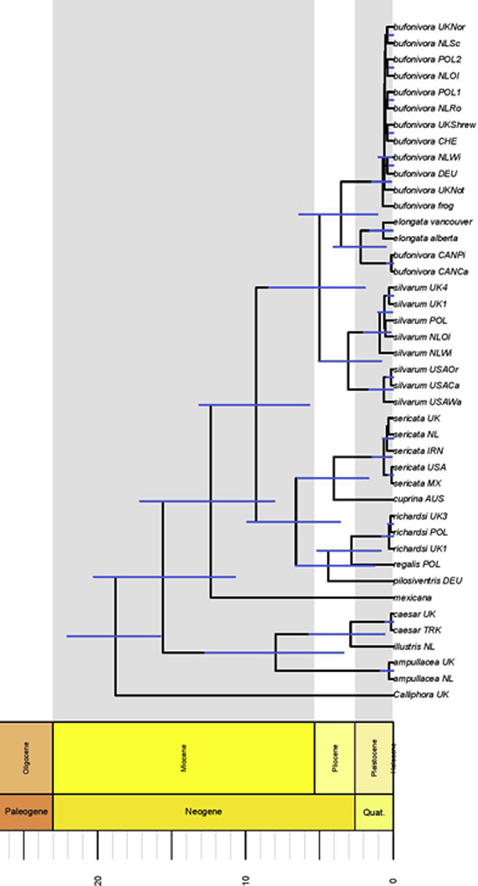

## 1 Introduction

Myiasis is the infestation of a living host, usually vertebrate, with dipterous larvae that feed on the tissues of the host (Zumpt, 1965). Within the super-family Oestroidea, many different lineages of flies are generally recognised as causing myiasis, ranging from highly specific obligate parasites to opportunistic facultative agents of myiasis. The family Calliphoridae includes a wide range of saprophagous, facultative myiasis agents and a small number of species of obligate parasites (Aubertin, 1933, Zumpt, 1965; Stevens et al., 2006), many of which are of major economic importance in the livestock industry (e.g. *Lucilia sericata*, *Lucilia cuprina*, *Cochliomyia hominivorax*). Most calliphorid flies typically exhibit low host-specificity, relatively short periods of larval development and are rarely seen infecting hosts in the wild (Zumpt, 1965; Erzinçlioğlu, 1989; Stevens et al., 2006). Thus, it has been hypothesized that *Lucilia* blowflies may have evolved facultative ectoparasitism in association with humans and animal domestication (Stevens and Wall, 1997a; Stevens et al., 2006). However, the toadfly, *Lucilia bufonivora*, exhibits obligate parasitism for amphibians and is generally associated with wild hosts that rarely survive infestation (Vestjens, 1958; Koskela et al., 1974; Strijbosch, 1980; Gosá et al., 2009; Díaz-Martín et al., 2012). The life history of facultative myiasis agents has been well studied in the past due to their economic importance as livestock parasites and as forensic indicators (Zumpt, 1965; Wall et al., 1992; Stevens and Wall, 1997a; Stevens, 2003; Wallman et al., 2005). Given the lack of economic importance of *L. bufonivora*, information on its evolutionary history is limited (Stevens and Wall, 1997a; Stevens, 2003). Moreover, precisely when *L. bufonivora* evolved this high host-specificity for amphibians is unknown and detailed phylogenetic studies are required to understand the evolution of obligate amphibian parasitism in a genus that is comprised mainly of saprophagous and facultative agents of myiasis.

Until recently, it was thought that *L. bufonivora* was a strictly Palearctic species; nonetheless, Tantawi and Whitworth (2014) recorded adult specimens for the first time in Canada. However, their study used only morphological characterisitcs and, to date, there are no published studies of the phylogenetic relationships between Nearctic and Palearctic populations of *L. bufonivora*. Moreover, although adult flies have been reported in North America, studies have not yet confirmed its involvement in amphibian myiasis in this region. Additionally, it is not known whether this constitutes a recent introduction to North America or simply reflects its relative rarity and/or previous taxonomic confusion.

In the United States and Canada, two other blowfly species have also been reported to be involved in amphibian myiasis: *Lucilia elongata* and *Lucilia silvarum* (Roberts, 1998; Bolek and Coggins, 2002; Bolek and Janovy, 2004). The former is restricted to the Nearctic and has never been observed breeding in carrion and, thus, it is also generally considered an obligate parasite of amphibians (Tantawi and Whitworth, 2014). In contrast, *L. silvarum* is distributed throughout the Holarctic (Rognes, 1991; Tantawi and Whitworth, 2014) and this species has been reported as being involved in amphibian myiasis in Europe (Duncker, 1891; Mortensen, 1892; Linder, 1924; Stadler, 1930). Nevertheless, a recent study found that in the UK, the Netherlands and Switzerland amphibian myiasis appears to be caused only by *L. bufonivora*, as no specimens of *L. silvarum* were found to be implicated in the disease (Arias-Robledo et al., 2019a). Moreover, the saprophagous behaviour of *L. silvarum* has been previously well documented (Hanski and Kuusela, 1977; Hanski, 1987; Prinkkila and Hanski, 1995; Fremdt et al., 2012). Nevertheless, blowflies often exhibit intraspecific behavioural differences according to their geographical region. As an example, the sheep blowfly *L. sericata* is typically a highly abundant saprophagous species in many countries, but behaves as a primary myiasis agent in Northern Europe (Wall et al., 1992; Wallman et al., 2005; Saloña-Bordas et al., 2009; Diakova et al., 2018). Hence, variation in the behaviour of *L. silvarum* and its involvement in amphibian myiasis may be possible in North America.

The mitochondrial gene cytochrome *c* oxidase subunit one (*COX1*) has proved to be a useful molecular marker for the detection and identification of various parasites and pathogens, including, but not limited to, nematodes (Aravindan et al., 2017), trypanosomes (Rodrigues et al., 2017), ticks (Chitimia et al., 2010) and oestrid flies (Samuelsson et al., 2013). Additionally, this mtDNA marker has provided clear resolution on the relationships of the *L. bufonivora* species group: *L. bufonivora*, *L. elongata* and *L. silvarum* (McDonagh and Stevens, 2011; Arias-Robledo et al., 2019a). In contrast, the various nuclear markers used to date have yielded mixed results and some are clearly not suitable for resolving the relationships of these recently diverged taxa (McDonagh and Stevens, 2011; Arias-Robledo et al., 2019a). Nonetheless, some other phylogenetic studies on Calliphoridae have shown that the use of optimised nuclear markers, such as the *period* gene (*per*) or the non-coding ribosomal DNA (rDNA) Internal Transcribed Spacer 2 (*ITS2*), are suitable for phylogenetic analysis of closely related blowfly taxa (Marinho et al., 2011; Williams and Villet, 2013). Thus, dependent on the nuclear DNA (nDNA) marker employed, some are apparently well suited for resolving relationships between *L. bufonivora*, *L. elongata* and *L. silvarum*.

The aims of this work were, firstly, to infer the times at which the life history trait of obligate amphibian parasitism arose within a genus that is mainly composed by species with sarco-saprophagous life cycles (*Lucilia*). To do this, the present work analysed samples from across the broad geographical range of *L. bufonivora*, *L. elongata* and *L. silvarum*. Molecular clock dating was performed using a concatenated data set comprising a nuclear (*per*), a mitochondrial (*COX1*) and a non-coding gene (*ITS2*). Secondly, this work aimed to define the degree of genetic divergence between Palearctic and Nearctic samples of *L. bufonivora* with the widely used mitochondrial marker *COX1*, whilst also solving the problem of the low phylogenetic resolution of this species group that has been previously encountered when using nuclear DNA markers. To do this, multiple phylogenies were inferred from sequence data obtained from optimised nuclear markers such as *per* and *ITS2*. Finally, molecular data were employed to determine whether *L. bufonivora* is involved in amphibian myiasis in North America.

In addressing these aims, this work also offers valuable information on the primers and PCR protocols needed for the successful amplification of a partial sequence of the protein-coding *per* gene of *L. bufonivora*. Additionally, we also provide sequence data for *Lucilia pilosiventris* and *Lucilia regalis,* blowfly species that have been understudied due to their relative rarity (Aubertin, 1933, Rognes, 1991; Szpila, 2017). The roles of geographical and ecological isolation on the speciation and evolution of blowfly species associated with amphibian myiasis are discussed.

## 2 Materials and methods

### 2.1 Biological material

**Fig. 1:**
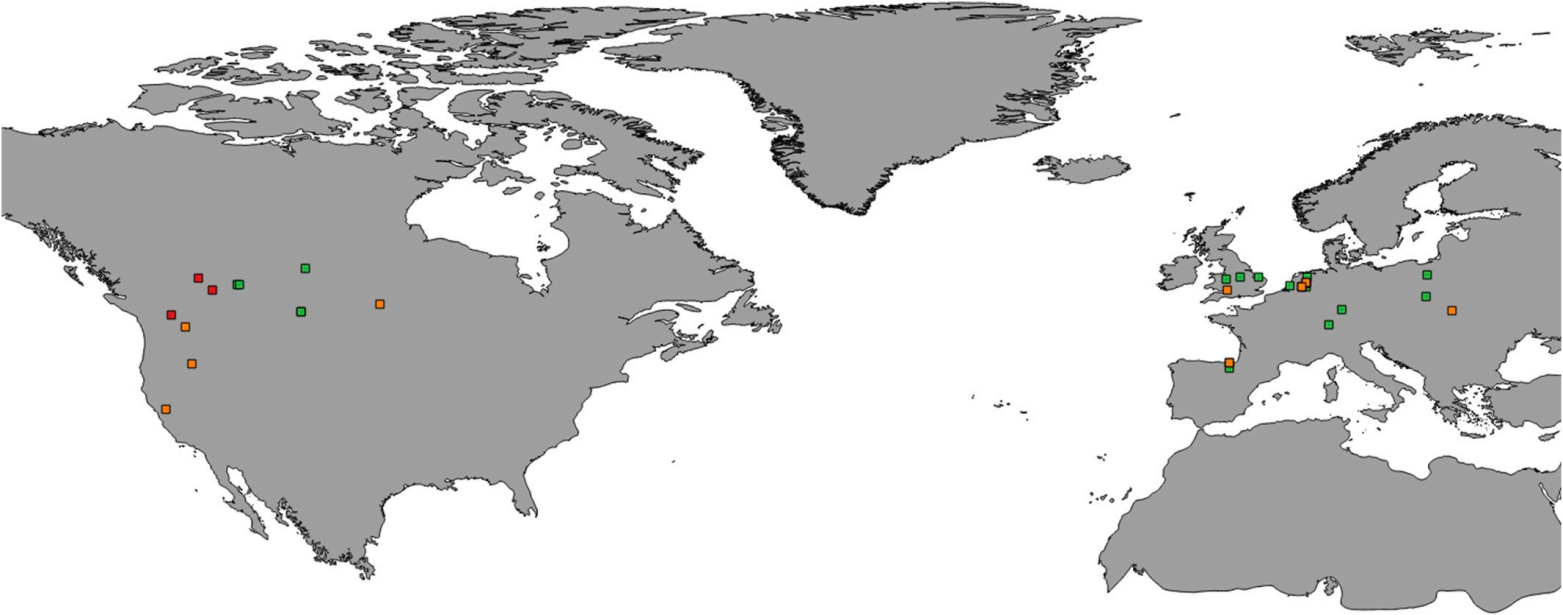
Location of samples for which the *COX1* gene was sequenced in this study. Boxes represent the locations of individual samples: red, *Lucilia elongata*; orange, *Lucilia silvarum*; green, *Lucilia bufonivora.*

Forty-two blowfly specimens were analysed in this study. Twelve specimens of *L. bufonivora* from different locations in Europe were included (Table 1). Additionally, two adult flies originally labelled as ‘*L. silvarum*’ that were reported to have caused amphibian myiasis in Alberta, Canada (Table 1) were identified morphologically as *L. bufonivora* using recent keys (Tantawi and Whitworth, 2014). These samples were also analysed and BLAST searches gave a 100% match with three *L. bufonivora COX1* sequences from Canada (Table 2); these sequences were also added to the data set along with an additional sequence from Spain (Table 2, Fig. 1).

**Table 1:**
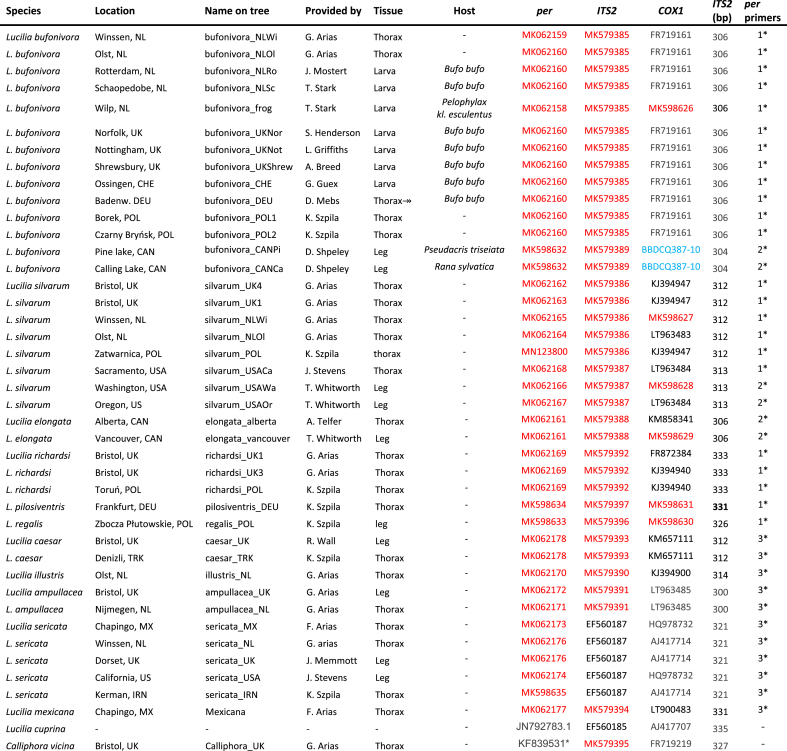
Specimen list. The table provides the location, name on tree, collector/provider, tissue used for DNA extraction, host (if any), their GenBank accession codes for their respective *per*, *ITS2* and *COX1* sequence data, length (bp) of *ITS2* sequences and primers used for the amplification of the *per* gene.

If no host listed, the samples were collected in its adult stage. Country abbreviations: NL = the Netherlands; UK= United Kingdom; CHE= Switzerland; DEU = Deustchland; POL = Poland; USA= United States of America; CAN=Canada; TRK = Turkey; MX = Mexico; IRN= Iran.

*per amplification primers: 1* = ***pbf*14 - *per*650-R** (present study); 2* = ***pbf*14 - *per*433-R** and ***pbf*249 - *per*650-R** (present study); 3* = ***per*5 - *per*reverse** (Williams and Villet, 2013).

Accession codes in blue belong to BOLD database. NOTE: Only new sequence data were submitted to GenBank as haplotypes (shown in red text), thus specimens with the same haplotype were allocated with the same accession codes.

**Table 2:** Additional COX1 sequences used in this study with their respective location, accession codes and public database where the sequences are available (BOLD/Genbank).

Species	Location	Accession Code	BOLD/GenBank
*Lucilia bufonivora*	Spain	GBDP15380-14	BOLD
*L. bufonivora*	Saskatchewan, CAN	BBDCQ387-10	BOLD
*L. bufonivora*	Saskatchewan, CAN	CNGSD7561-15	BOLD
*L. bufonivora*	Saskatchewan, CAN	MF758767.1	GenBank
*Lucilia silvarum*	Spain	KJ394941.1	GenBank
*L. silvarum*	Manitoba, CAN	SMTPR3630-16	BOLD
*Lucilia elongata*	Vancouver, CAN	BBDCP287-10	BOLD
*L. elongata*	Washington, USA	GMNCF036-12	BOLD
*Lucilia thatuna*	Callifornia, USA	BBDIT928-11	BOLD
*L. thatuna*	San Francisco, USA	DQ453489	GenBank
*Lucilia richardsi*	Germany	GMGMA838-14	BOLD

Eight adult specimens of *L. silvarum* were analysed. Five were collected from different locations in Europe and three from the USA. Two *COX1* sequences obtained from BOLD and GenBank (from Canada and Spain, respectively) were also included in the analysis (Table 2).

Compared with other blowfly species in North America, *Lucilia elongata* is rarely encountered in the field. This study obtained one specimen from Vancouver, Canada and another from Alberta (Table 1, Fig. 1). Two additional *COX1* sequences from the United States and Canada were obtained from BOLD and included in the analysis (Table 2).

Phylogenetic relationships between the sheep blowflies (*L. sericata* and *L. cuprina*) have been well studied in the past due to their economic importance (Stevens and Wall, 1997b; Wallman et al., 2005; Williams and Villet, 2013). For comparative reasons, this study analysed five *L. sericata* specimens from a broad geographical range (Mexico, United States, The Netherlands, Iran and the UK; Table 1). All *L. cuprina* sequence data were obtained from Genbank (Table 1).

Specimens of *Lucilia ampullacea, Lucilia caesar*, *Lucilia illustris*, *Lucilia mexicana, Lucilia richardsi*, *L. regalis* and *L. pilosiventris* were also included in the analysis (Table 1). A specimen of *Calliphora vicina* from Bristol, UK, was used in the analyses as an outgroup; *per* gene sequence data for *C. vicina* were obtained from GenBank (KF839531). Finally, two additional *COX1* sequences of *Lucilia thatuna*, another species believed to be implicated in amphibian myiasis in North America (Tantawi and Whitworth, 2014), were included in the analysis (Table 2).

### 2.2 DNA extractions, primer design and polymerase chain reaction (PCR) procedures

Where possible, to avoid contamination, thoracic muscle fibres were extracted from whole adult specimens and used for extractions. With rare insect collection material, DNA extraction was undertaken from single legs. For this, muscle fibres were extracted from the trochanter, femur, tibia and, if available, the coxa. This was done by dissecting the legs in ethanol with the aid of a sterile scalpel blade and entomological pins. In the case of larval specimens, anterior and posterior parts of the larvae were used (or the whole specimen if it was a 1st stage larva). DNA extractions were carried out using a QIAGEN DNeasy ® Blood and Tissue Kit (Qiagen GmbH, Germany) according to the manufacturer's instructions.

When DNA extraction was undertaken from a single leg, once the tissue was extracted it was put in a mix of 80 μL of ATL buffer and 20 μL of Proteinase-K. Cell lysis was carried overnight at 56 °C. In order to increase the yield and concentration of extracted DNA, 40 μL of elution buffer (EB) were added to the spin-column and it was held for 30 min before the spin-down. DNA templates were stored at −20 °C until required. Prior to PCR, the concentration of DNA of each template (ng/μL) was checked using a NanoDrop One spectrophotometer (Thermo Scientific).

Amplification of the protein-coding *per* gene from various *Lucilia* species was carried out using the primers of Williams and Villet (2013). However, these primers did not prove suitable for the amplification of this gene in *L. bufonivora*. Therefore, a new set of primers (pbf14 and pbf650-R, Table 3) was designed for the amplification of ~610 bp of the nuclear protein-coding gene *per* in the *L. bufonivora* species group. This procedure was carried out using the online software Primer3 v 3.4 (Untergasser et al., 2007). and by ensuring that the difference in melting point (TM) between primers was less than 0.5 °C and that each primer had a Guanine-Cytosine base content of at least 50%. In the case of single leg extractions, an additional set of primers was designed in order to amplify the partial sequence of the *per* gene in two overlapping fragments, each of ~410 bp (*pbf*14 + *p*433-R and *p*249 + *pbf*650-R; Table 3). All primer sequences and PCR protocols for the amplification of *COX1* and *ITS2* are listed in Table 3.

**Table 3:** Primers used for the amplification of *per*, *COX1* and *ITS2*. Name, sequence, source and PCR protocols are described.

Gene	Name	Sequence	Source	Protocol					
				ID	D	A	E	C	F
*per*	*per*5	GCCTTCAGATACGGTCAAAC	Williams and Villet (2013)	94 °C 5min	94 °C 30s	50 °C 1min	72 °C 30s	x36	72 °C 7min
	*per*reverse	CCGAGTGTGGTTTGGAGATT							
	*pbf*14	GGCGTTGTCAAGCTCTAGC	this study	94 °C 5min	94 °C 30s	48 °C 1min	72 °C 30s	x36	72 °C 7min
	*pbf*650-R	CCACGAATGTGAACCAACTC							
	*p*249	GCAAACCAGTAACAGCACCT							
	*p*433-R	GTGCCTGTACCGGTGTTG							
*COX1*	LCO1490	GGTCAACAAATCATAAAGATATTGG	Folmer et al. (1994)	94 °C 5min	95 °C 30s	45 °C 30s	72 °C 1min	x35	72 °C 7min
	HCO2198	TAAACTTCAGGGTGACCAAAAAATCA							
*ITS2*	ITS4	TCCTCCGCTTATTGATATGC	White et al. (1990)	*94 °C 2min	94 °C 30s	44 °C 35s	72 °C 30s	x38	72 °C 3min
	ITS5.8	GGGACGATGAAGAACGCAGC							

*ID = initial denaturation step, D = denaturation, A = annealing, E = extension, C = cycles of D-A-E, F = final extension.

The *ITS2* is a non-coding nuclear ribosomal RNA subunit located between the 5.8S and 28S ribosomal subunit DNAs. To amplify the complete *ITS2* sequence, primers were located in the 3′ end of the 5.8S subunit and the 5′ end of the 28S subunit, as described by Marinho et al. (2011). Additionally, in Calliphoridae, another small subunit (2S) splits the *ITS2* region in two fragments: *ITS2a* (~30 bp) and *ITS2* (300–335 bp). Of these, *ITS2a* has minimal sequence variation and was excluded from further analysis, while amplification of the longer *ITS2* region exhibited very variable sequence length among the taxa studied (Table 1).

PCR products were purified using 0.5 μL of exonuclease and 0.5 μL of Antarctic phosphatase per 20 μL of PCR product. After purification, both forward and reverse strands were sequenced by a commercial sequencing facility, EUROFINS®.

New sequence data were submitted to GenBank as haplotypes; specimens with the same haplotype were allocated the same accession code (Table 1).

### 2.3 Sequence editing and alignment

Forward and reverse chromatograms were checked manually for potential reading errors using the BioEdit software (Hall, 1999). This software was also used for assembling both strands into a single consensus sequence. Consensus sequences were subjected to BLAST searches to confirm species identity. Alignment was done using the ClustalW algorithm in BioEdit.

In the case of heterozygous sequences (*per*), both forward and reverse chromatograms were checked using BioEdit. Sites that presented two different nucleotide peaks within the same site and with the same height were considered as heterozygous sites. Consensus sequences were encoded using their respective IUPAC annotation.

### 2.4 Phylogenetic analyses

Firstly, single-gene phylogenies were reconstructed to illustrate the different mutation rates exhibited in mtDNA (*COX1*), nDNA (*per*) and non-coding rDNA (ITS2). Substitution model selection for single-gene data sets was carried out using jModeltest (Posada, 2008); the best-fitting model was chosen using the Bayesian Information Criterion (*ITS2*) and the Akaike Information Criterion (*per*, *COX1*). The models selected were: GTR + F + I + G4 for *COX1*; TIM2+G for *per*; and K3Pu + F + G4 for *ITS2*. In the *ITS2* data set, gaps were treated as complete deletions. Bayesian inference analysis was done with the software MrBayes v3.2.6 (Huelsenbeck and Ronquist, 2001) by implementing the corresponding substitution model with each data set. A Markov Chain Monte Carlo (MCMC) method was used, starting from two simultaneous independent runs, with three heated chains and one cold chain. Each was run for 10 million generations, sampling every one thousand generations. When the critical value for the topological convergence diagnostic fell below the default threshold (0.01) analyses were stopped. Burn-in was set to 0.25 to discard a fraction of sampled values. Trees were drawn using R in Rstudio (Team R., 2015) with the package ggtree (Yu et al., 2017). Pairwise distances for *COX1* were calculated using MEGA7 (Kumar et al., 2006). In cases of sequence heterozygosity (*per* gene), sequences were formatted in SeqPHASE (Flot, 2009) and alleles were inferred using PHASE under the default settings.

To provide a clearer resolution of the evolutionary relationships of the *L. bufonivora* group using nuclear DNA, a parsimony splits network based on a concatenated data set with the inferred *per* alleles and the non-coding *ITS2* was drawn under the default conditions of SplitsTree (Huson and Bryant, 2006).

### 2.5 Divergence time estimation

Tree calibration was done by specifying the node age corresponding to the split between the Luciliinae and Calliphorinae subfamilies (19.7 mya), as estimated by Wallman et al. (2005). Sequence data for the three genes (mtDNA, nDNA and non-coding rDNA) were used for this analysis. Best-fit substitution models were unlinked to allow different evolution rates. Additionally, to allow substitution rates to vary among lineages, the clock model was set to an unlinked log-normal relaxed clock for each gene separately. Clock rate was set to ‘estimate’ for each data set under BEAST (Suchard et al., 2018) default settings. MCMC consisted of two independent runs, each with a sampling size of 20 million, with samples logged every 1000 steps. Convergence between runs was checked using Tracer. Tree files were combined using LogCombiner with a burn-in set to 10%. The software TreeAnnotator from the BEAST package, was used for annotating the maximum credibility tree. The latter was drawn using the package ‘strap’ (Bell and Lloyd, 2014) using R in Rstudio (Team R., 2015).

## 3 Results

In summary, amphibian parasitism was recovered as a monophyletic life history trait in all phylogenies inferred. The saprophagous species *L. silvarum* was never included in this monophyletic group. Samples of the toadfly *L. bufonivora* showed consistent paraphyly with respect to *L. elongata*, showing a clear distinction between individuals from Europe and Canada. The well-recognised relationships between the sheep blowflies *L. sericata* and *L. cuprina* were recovered with strong support in all phylogenies. Similarly, the *L. caesar* species group, comprised mainly of saprophagous species with very similar morphology, was supported by strong PPO values in all phylogenies.

### 3.1 Single-gene phylogenies

**Fig. 2:**
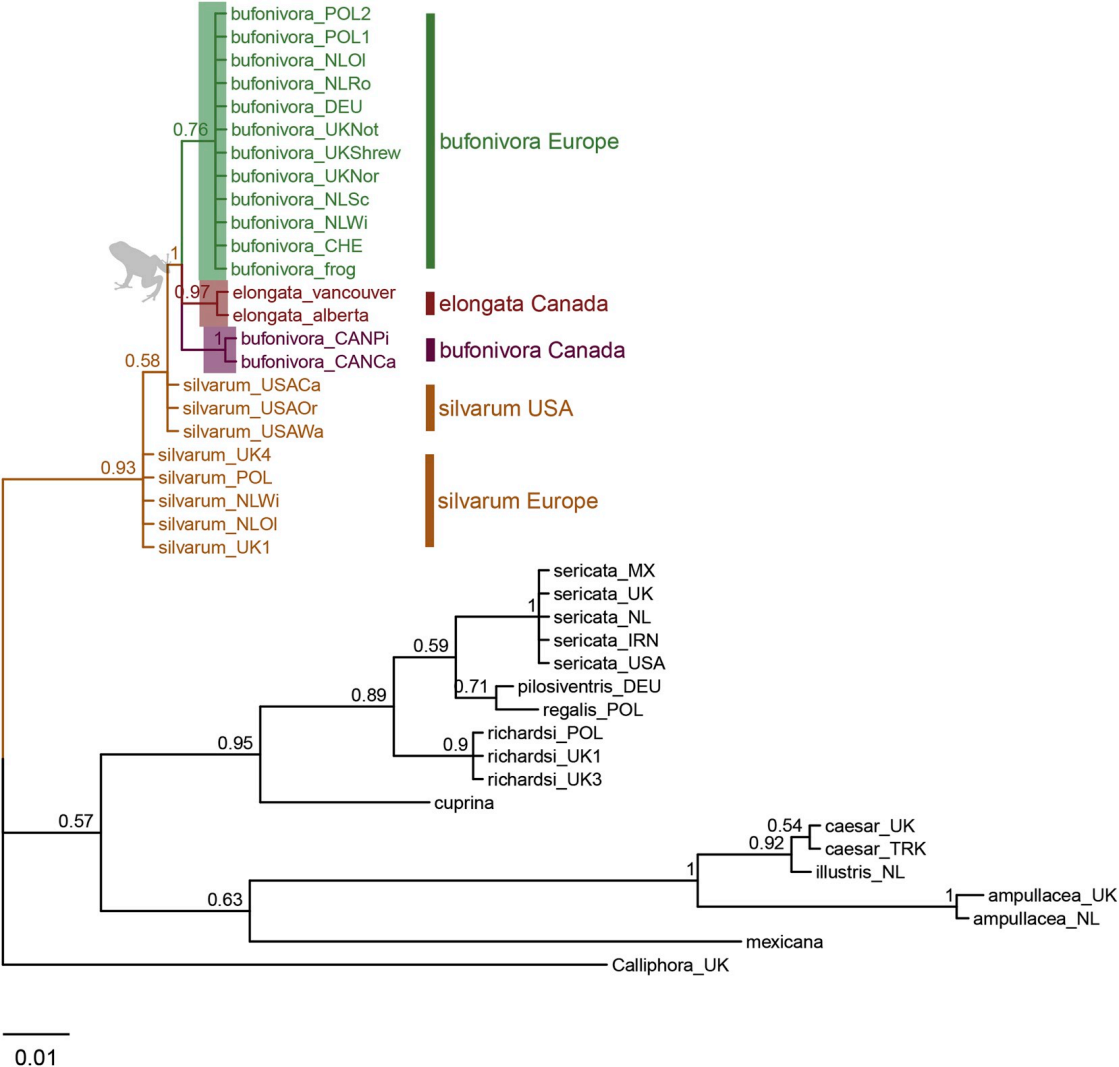
Bayesian Inference tree constructed from Internal transcribed Spacer 2 (non-coding) sequence data. Each specimen is labelled with the species name and location abbreviation as indicated in Table 1. Green text corresponds to European samples of *Lucilia bufonivora*; red represents *Lucilia elongata*; purple represents Canadian *L. bufonivora*; orange represents *Lucilia silvarum.* Scale bar represents expected changes per site. (For interpretation of the references to colour in this figure legend, the reader is referred to the Web version of this article.)

The *ITS2* sub-region exhibited very variable sequence length among taxa (Table 1). Accordingly, phylogenetic analysis of the *ITS2* subunit included sequence data for the *2S* (partial), *ITS2* and *28S* (partial) regions. European sequences of *L. bufonivora* exhibited a consistent haplotype with the presence of an 8 bp indel that was not observed in the Canadian haplotype of the same species. This tree supported the paraphyly of *L. bufonivora* with respect to *L. elongata* (Fig. 2). Whilst generally exhibiting lower posterior values, this tree recovered a European *L. silvarum* clade that was distinct from a North American clade of the same species (Fig. 2). Unlike *L. bufonivora*, all samples of the sheep blowfly, *L. sericata*, were recovered in a monophyletic clade regardless of the geographical distances between them. Similarly, samples of *L. caesar* from Turkey and the UK did not exhibit any intraspecific variation within this phylogeny (Fig. 2).

**Fig. 3:**
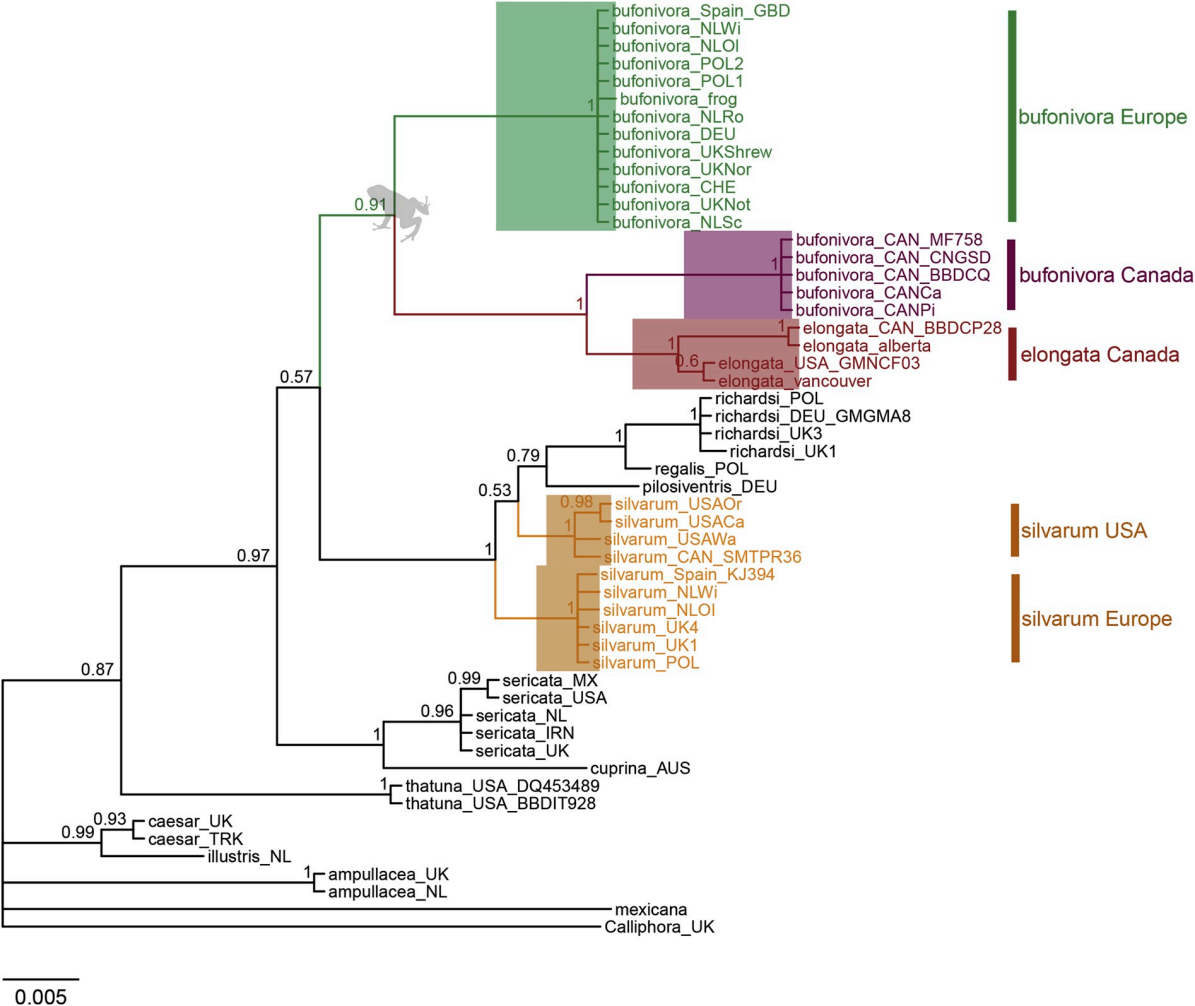
Bayesian Inference tree constructed from *COX1* (mtDNA) sequence data. Each specimen is labelled with the species name and location abbreviation as indicated in Table 1. Sequences obtained from BOLD/GenBank are also annotated with their respective accession codes. Green text corresponds to European samples of *Lucilia bufonivora*; red represents *Lucilia elongata*; purple represents Canadian *L. bufonivora*; orange represents *Lucilia silvarum*. Scale bar represents expected changes per site. (For interpretation of the references to colour in this figure legend, the reader is referred to the Web version of this article.)

Bayesian inference analysis of the *COX1* data set suggested a rapid mutation rate in the mtDNA of the *L. bufonivora* species group (Fig. 3). All Canadian samples of *L. bufonivora* were clustered together in a single clade independent from a European clade. Thus, *L. bufonivora* was defined as paraphyletic with respect to the strictly Nearctic *L. elongata.* Indeed, Canadian samples of *L. bufonivora* appear to have a closer affinity with *L. elongata* than with their European conspecifics (Fig. 3); certainly, they exhibited relatively high intraspecific genetic distances (0.050–0.052, Table 4). Similarly, within this phylogeny, *L. silvarum* was recovered as a paraphyletic species with respect to *L. richardsi*/*L. pilosiventris*/*L. regalis* (Fig. 3). Although *L. thatuna* has previously been considered as being implicated in amphibian myiasis in North America (Tantawi and Whitworth, 2014), it does not appear to have close relationships with the *L. bufonivora* species group (Fig. 3). Within this phylogeny the Australian sheep blowfly, *L. cuprina*, grouped next to the *L. sericata* clade with strong support (Fig. 3). Surprisingly, the pairwise distance displayed between them was lower than that observed between Canadian and European *L. bufonivora* (0.022–0.024, Table 4).

**Table 4:**
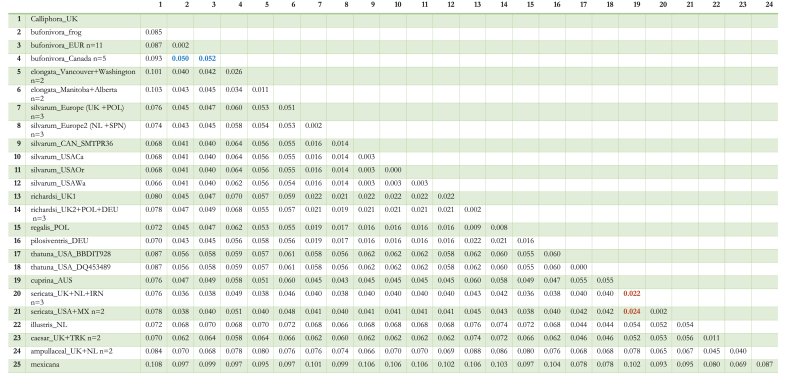
The pairwise genetic distances computed with *COX1* sequence data of various *Lucilia* specimens. Numbers in blue highlight the distance between European and Canadian *Lucilia bufonivora.* Letters in red highlight the distance between *Lucilia sericata* and *Lucilia cuprina*.

*n = number of sequences with the same haplotype.

**Fig. 4:**
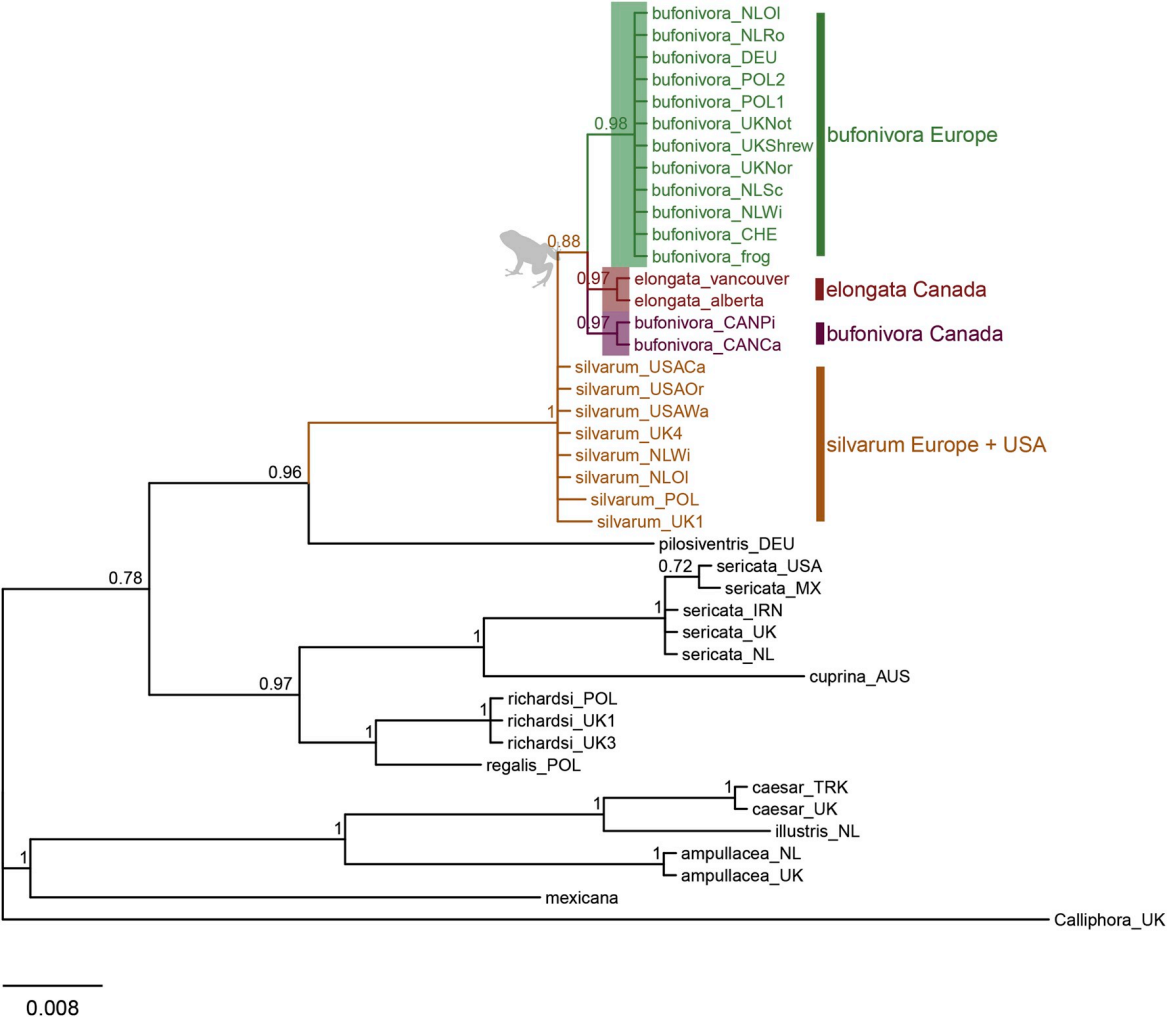
Bayesian Inference tree constructed from *per* gene (nDNA) sequence data. Each specimen is labelled with the species name and location abbreviation as indicated in Table 1. Green text corresponds to European samples of *Lucilia bufonivora*; red represents *Lucilia elongata*; purple represents Canadian *L. bufonivora*; orange represents *Lucilia silvarum.* Scale bar represents expected changes per site. (For interpretation of the references to colour in this figure legend, the reader is referred to the Web version of this article.)

The single gene phylogeny inferred from the *per* (nDNA) gene also supported the paraphyly of *L. bufonivora* with respect to *L. elongata* (Fig. 4). Unlike previous phylogenies, all samples of *L. silvarum* (both European and North American) were grouped in a single clade with strong support (Fig. 4).

Within the *ITS2* and *COX1* phylogenies, *L. richardsi, L. pilosiventris* and *L. regalis* showed close relationships to one another (Fig. 2, Fig. 3). These species are morphologically similar to the sheep blowfly *L. sericata*, which is in agreement with the *ITS2* phylogeny (Fig. 2). Nevertheless, within the *COX1* phylogeny, these species were recovered as a sister group to the North American *L. silvarum* clade (Fig. 3).

### 3.2 Parsimony splits: *per + ITS2*

**Fig. 5:**
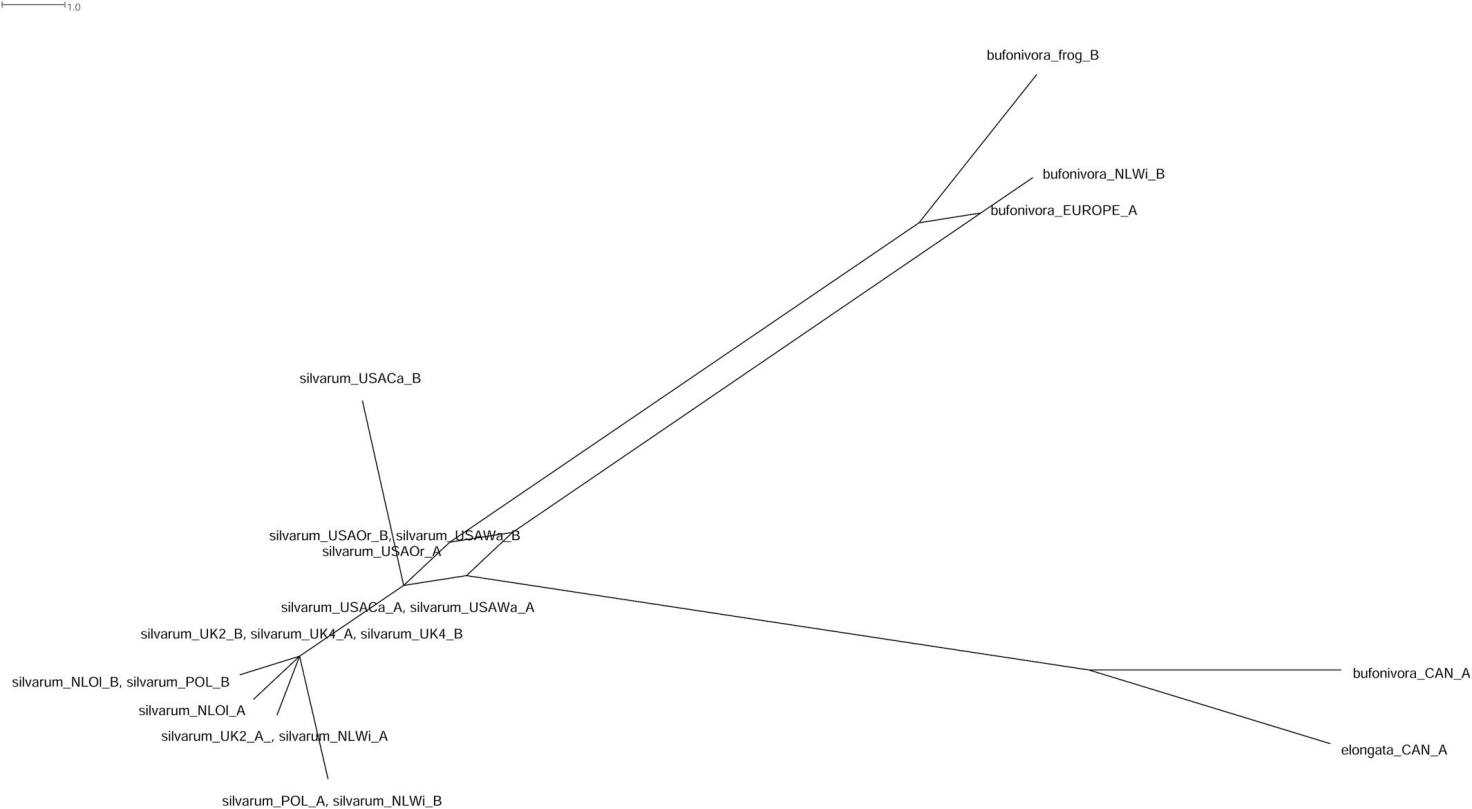
Parsimony splits network constructed from a *per* and *ITS2* concatenated sequence data set. Heterozygous specimens are indicated with A and B. ‘bufonivora_EUROPE_A’ represents a consistent haplotype present in all 12 samples from Europe (Table 1), of which just two were heterozygous (‘bufonivora_frog’ and ‘bufonivora_NLWi’). ‘bufonivora_CAN’ and ‘elongata_CAN’ are represented by two samples each, none of which were heterozygous. Scale bar represents expected changes per site.

A concatenated data set of *per* and *ITS2* gene sequence data allowed the analysis of ~1050 bp of sequence. The resulting parsimony splits network showed better resolution using nDNA than those based on single-gene phylogenies (Fig. 2, Fig. 4). As suggested previously by the *COX1* phylogeny, parsimony splits of the concatenated data set produced two well-separated groups of amphibian parasites: a Nearctic (*L. elongata* and Canadian *L. bufonivora*) and a Palearctic (European *L. bufonivora*) group. Both displayed almost the same genetic distance with respect to the *L. silvarum* sister cluster (Fig. 5).

### 3.3 Divergence time estimation

**Fig. 6:**
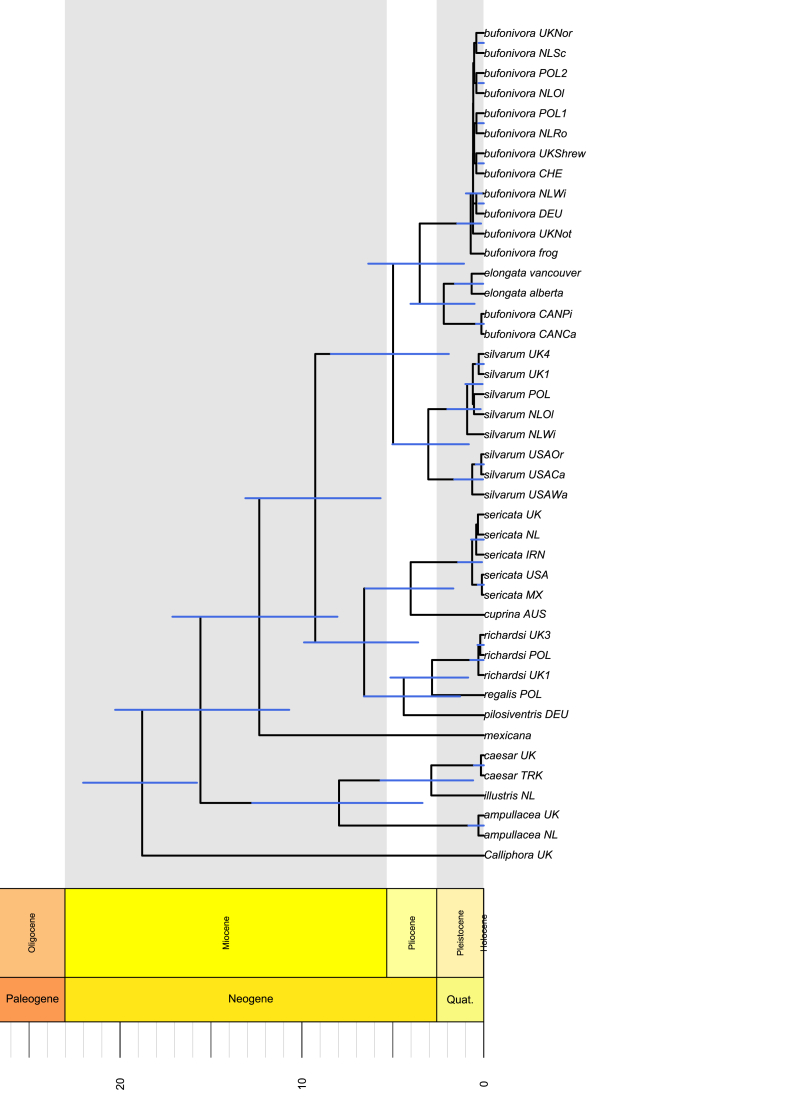
Divergence times estimated from a concatenated data set of *per*, *COX1* and *ITS2* sequences for the *Lucilia bufornivora* species group. Substitution model and relaxed clock models were unlinked for each gene. The tree was calibrated by setting the root to the node age corresponding to the split between Luciilinae and Calliphorinae subfamilies (~19 mya) as estimated by Wallman et al. (2005). Blue bars represent 95% highest posterior density (HPD) of each node age. (For interpretation of the references to colour in this figure legend, the reader is referred to the Web version of this article.)

A concatenated data set of *COX1*, *ITS2* and *per* (~1700 bp) was analysed. A Bayesian uncorrelated relaxed clock was used to estimate the divergence times for a range of different species of *Lucilia*. The molecular clock calibration was set to the split between the subfamilies Luciliinae and Calliphorinae, which has been estimated to have happened around 19.7 mya (Wallman et al., 2005). The present estimates indicate that the main radiation of the genus *Lucilia* occurred during the middle Miocene, about 15.57 mya (95% CI: 10.69–20.26 mya, Fig. 6). Our results suggest that during this time, there was a major split between a lineage of species with predominantly saprophagous habits (the *L. caesar* group) and a lineage that ultimately would include the sheep blowfly (*L. sericata*) and the toadfly (*L. bufonivora*) species groups (Fig. 6).

The split between the *L. bufonivora* and the *L. sericata* species groups was inferred to have occurred during the Miocene, around 9.26 mya (95% CI: 5.60–13.10 mya, Fig. 6). This suggests that the *L. bufonivora* group may have diverged from a saprophagous/facultative ancestor. Diversification of the *L. bufonivora* group was estimated to have occurred during the Pliocene Epoch, 4.98 mya (CI: 1.92–8.40 mya, Fig. 6). Within this group, niche isolation of their most recent ancestor may have played an important role in the adaptative radiation of two distinct lineages: one with saprophagous behaviour (*L. silvarum*) and another that evolved high host-specificity for amphibians (*L. bufonivora* + *L. elongata*).

Similarly, the divergence between a Nearctic and Palearctic *L. silvarum* was inferred to occur around 3.05 mya (95% CI: 0.80–5.02 mya, Fig. 6). Thus, this finding suggests the independent evolution of this saprophagous species in two geographically isolated populations.

## 4 Discussion

### 4.1 Phylogenetic relationships

Previous studies have suggested that the parasitic habit in *Lucilia* blowflies evolved independently on multiple occasions (Stevens and Wall, 1997a; Stevens, 2003; Stevens and Wallman, 2006). Present results support this hypothesis, showing a clear distinction between the different *Lucilia* species groups, most of which include taxa that exhibit both saprophagous and parasitic life histories. In contrast, however, obligate parasitism and specialisation for a distinct host species group (amphibians) appear to have evolved just once, as indicated by the reciprocal monophyly of *L. bufonivora* and *L. elongata* (Fig. 2, Fig. 3, Fig. 4). Moreover, while *L. silvarum* showed close relationships with the latter two species, it was never incorporated into the monophyletic group of taxa associated with obligate amphibian myiasis. This is finding is perhaps to be expected given that *L. silvarum* is a well-documented saprophage (Hanski and Kuusela, 1977; Hanski, 1987; Prinkkila and Hanski, 1995; Fremdt et al., 2012).

Mutation rates in mtDNA are generally faster than those in nuclear DNA due to the lack of recombination and the accumulation of deleterious mutations (Brown et al., 1979; Neiman and Taylor, 2009). Within recently diverged species of *Lucilia* blowflies, this can result in shorter branches in nuclear phylogenies but longer branch lengths in mtDNA-based phylogenies (McDonagh and Stevens, 2011; Yusseff-Vanegas and Agnarsson, 2017). Certainly, the single-gene phylogenies presented here showed an accelerated mtDNA mutation rate within the *L. bufonivora* species group. This has also been reported in other insect groups, such as crabronid wasps (Hymenoptera) (Kaltenpoth et al., 2012).

All phylogenies exhibited a clear distinction between well-defined clades of Nearctic and Palearctic *L. bufonivora.* Evidence from the concatenated time-scaled phylogeny and parsimony splits networks (*ITS2* + *per*) suggests that *L. bufonivora* from Canada has greater affinity with the strictly Nearctic *L. elongata* than with its Palearctic conspecifics. Surprisingly, the *COX1* intraspecific pairwise distance between Canadian and European individuals of *L. bufonivora* was greater than the interspecific distance displayed between the sheep blowflies *L. sericata* and *L. cuprina.* Thus, geographical isolation of *L. bufonivora* and rapid mtDNA evolution rates appear to be facilitating on-going cryptic speciation. This phenomenon is relatively common within Diptera, as reported in geographically isolated populations of gall midges, tephritid flies, flesh flies and black flies (Hall et al., 2009; Tadeo et al., 2015; Adler et al., 2016; Duque-Gamboa et al., 2018). The status of *L. bufonivora* in Canada as a distinct species, however, remains to be resolved, and will also require detailed morphological examination of specimens from both Eastern and Western hemispheres.

Phylogenetic resolution of the *L. bufonivora* species group is often poor when using nuclear DNA markers (McDonagh and Stevens, 2011; Arias-Robledo et al., 2019a). In fact, a recent study failed to differentiate *L. elongata* from *L. silvarum* using the gene *EF1α*, highlighting the close relationships of this species group (Arias-Robledo et al., 2019a). In the present study, single-gene phylogenies inferred from both *per* and *ITS2* sequence data provided clearer resolution on the relationships of this species group and recovered *L. elongata* as being closely related to *L. silvarum*. Furthermore, the parsimony splits from a concatenated data set of the aforementioned genes indicated a clear split between a Nearctic lineage (Canadian *L. bufonivora* and *L. elongata*) and a Palearctic (European *L. bufonivora*) grouping of obligate parasites of amphibians that exhibit almost the same genetic distance with respect to *L. silvarum*. All taxa from the *L. bufonivora* species group exhibited unique and consistent *ITS2* haplotypes with differences in length and base composition. Therefore, unambiguous species identification can be carried out employing multi-locus analysis with *COX1* and *ITS2* sequence data (Jordaens et al., 2013; GilArriortua et al., 2014; Yusseff-Vanegas and Agnarsson, 2017).

The saprophagous species *L. silvarum* exhibited high mtDNA sequence divergence between Palearctic and Nearctic samples. While this result could be viewed as indicative of species-level differentiation, it is suggested that this should be interpreted with caution. For instance, unlike for *L. bufonivora*, the concatenated time-scaled tree recovered *L. silvarum* as monophyletic (Fig. 6). Similarly, previous molecular studies on other blowflies, e.g., *Phormia regina*, have detected high mtDNA sequence divergence between North American and European populations (Desmyter and Gosselin, 2009; Boehme et al., 2012). Due to a lack of morphological differentiation and minimal nuclear DNA variation, it was concluded that the mtDNA variation observed in *P. regina* did not indicate species-level differentiation (Jordaens et al., 2013). This phenomenon has also been reported for other species such as *Lucilia eximia* and *Lucilia rica* (Yusseff-Vanegas and Agnarsson, 2017). In addition, in the current study, Bayesian analysis of *per* gene data clustered Nearctic and Palearctic *L. silvarum* in a single clade. And, in contrast with *L. bufonivora*, the parsimony splits network also clustered all samples of *L. silvarum* close to each other (Fig. 5). These findings suggest that *L. silvarum* also exhibits an accelerated mtDNA mutation rate; thus, accelerated mtDNA evolution might have been present in the saprophagous ancestor of *L. bufonivora* and cannot be attributed directly to its highly specific life history.

It is well recognised that multi-locus phylogenies typically provide deeper insights into the evolutionary history of an organism than do single-gene phylogenies (Wallman et al., 2005; McDonagh and Stevens, 2011). Nonetheless, the latter can still be useful to illustrate potential differences in mutation rates exhibited by individual loci. Single-gene trees may also help in detecting ancient hybridisation and/or incomplete lineage sorting events. Certainly, the current study found that the less commonly encountered species *L. richardsi/L. pilosiventris/L.regalis* comprise a species group, which, in turn, is related to both *L. sericata* (*ITS2*, Fig. 2) and *L. silvarum* (*COX1*, Fig. 3). To date, only one morphology-based phylogenetic study has recovered a close relationship between *L. regalis/L. pilosiventris* and the saprophage *L. silvarum* (Stevens and Wall, 1996). Although in the *COX1*-based mtDNA phylogeny presented here (Fig. 3) the relationship between these taxa accords with that presented by Stevens and Wall (1996), the positioning of these taxa within the *ITS2* phylogeny is in marked contrast, with *L. pilosiventris/L. regalis* and *L. richardsi* being grouped more closely with *L. sericata* (Fig. 2). In the past, this incongruency has been detected only for *L. richardsi* (McDonagh and Stevens, 2011; Arias-Robledo et al., 2019a). In *Drosophila* spp., such incongruencies are attributed to incomplete lineage sorting (Pollard et al., 2006) and we suggest a similar explanation for the incongruence observed between the mtDNA and nuclear DNA phylogenies in the current study. For example, in the current study the toadfly species group (*L. bufonivora*/*L. silvarum*/*L. elongata*) and the sheep blowfly species group (*L. sericata*/*L. richardsi*/*L. regalis*) share a common ancestor (Fig. 3, Fig. 4, Fig. 6). Thus, it is likely that after the rapid speciation of the ancestral form, polymorphisms were fixed randomly in each species (e.g. *L. sericata* and *L. bufonivora*) and, in some cases involving non-sister species, this could have resulted in the fixation of the same ancestral polymorphisms (e.g. in *L. richardsi* and *L. silvarum*). Nonetheless, further studies with more loci and/or mitogenomic data are required to confirm this hypothesis.

### 4.2 Evolution of obligate parasitism in *Lucilia* blowflies and specificity for amphibian hosts

The time-scaled phylogeny (Fig. 6) suggests that the diversification of genera within Calliphoridae seems to have occurred ~15.57 mya (95% CI: 10.69–20.26 mya), which is in accordance with several previous estimates (Wallman et al., 2005; Junqueira et al., 2016). Nonetheless, it has been suggested that some economically important calliphorid flies (i.e. *L. sericata* and *L. cuprina*) may have evolved parasitic behaviour in association with humans and the domestication of animals, as myiasis is rarely reported in wild animals (Erzinçlioğlu, 1989; Stevens and Wall, 1997a). However, high host-specificity for wild amphibians suggests that *L. bufonivora* evolved independently from those blowfly species associated with animal domestication. Indeed, the time-scaled phylogeny suggests that this life history trait arose approximately 5 mya, during the Early Pliocene (Fig. 6). In some groups of strictly obligate taxa such as oestrid flies, host-parasite coevolution appears to have played an important role in lineage divergence and speciation (Pape, 2006; Stevens et al., 2006). This, however, differs considerably from the evolution of *L. bufonivora*, which shows close phylogenetic affinity with other fly species that exhibit predominantly saprophagous feeding habits (e.g. *L. silvarum*).

The close relationship of *L. bufonivora* with *L. silvarum* suggests that their last common ancestor probably exhibited facultative parasitism or saprophagous feeding habits. Moreover, this idea is also supported by the reciprocal monophyly of the toadfly (*L. bufonivora*) and the sheep blowfly (*L. sericata*) species groups, both of which are comprised of parasitic and saprophagous taxa. Our results suggested that the split between these two species groups occurred in the Miocene around 9.26 mya (95% CI: 5.60–13.10 mya, Fig. 6). The time-scaled phylogeny also suggests that the saprophagous ancestor of *L. bufonivora* may have co-existed with other calliphorid lineages that behaved mostly as carrion-breeders (e.g. *Calliphora*, Fig. 6). It is well known that ephemeral resources, as provided by carrion, can facilitate intense interspecific competition (Hanski and Kuusela, 1977; Hanski, 1987; Prinkkila and Hanski, 1995). Intense competition within the carrion fly community may have forced the saprophagous ancestor of *L. bufonivora* to migrate to narrower ecological niches with fewer competitors; it may have started by infesting already injured amphibian hosts and/or colonising toad carcasses within minutes after death, thus facilitating in evolutionary time an adaptative radiation of a lineage of obligate parasites, namely *L. bufonivora*, and a saprophagous lineage that remained active in the carrion fly community (*L. silvarum*). The monophyletic origin of obligate amphibian parasitism in *Lucilia* blowflies is in marked contrast with the evolution of obligate parasitism of mammals, which appears to have had multiple independent origins in the Calliphoridae (Stevens, 2003; McDonagh and Stevens, 2011).

In contrast to *L. bufonivora,* the sheep blowfly, *L. sericata*, is a common and highly abundant species in many parts of Europe, including the UK (Rognes, 1991; Hwang and Turner, 2006; Arias-Robledo et al., 2019b). The large population sizes, high migration capacity and fertility of *L. sericata* are reported to have increased rates of gene flow and to have reduced the impact of genetic drift (Diakova et al., 2018). This would explain the genetic similarity of many of the geographically distant samples of *L. sericata* included in this study; moreover, this finding accords with previous research showing minimal intraspecific variation in much larger and spatially broader samples of *L. sericata* (Stevens and Wall, 1997b; DeBry et al., 2010; McDonagh and Stevens, 2011; Williams and Villet, 2013). In contrast, the low abundance of *L. bufonivora* in the field suggests that small population sizes, in combination with a restricted dispersal capacity, make the toadfly a species vulnerable to genetic drift, thereby facilitating the rapid independent evolution of geographically isolated populations, resulting in the high genetic distances observed between Nearctic and Palearctic *L. bufonivora*. A similar finding has been reported in a non-synanthropic flesh fly involved in obligate myiasis of different mammal species, *Wohlfahrtia vigil* (Hall et al., 2009). Given that *W. vigil* does not affect livestock host species, it is unlikely to have been dispersed by human activity, thus, it has evolved independently in the Eastern and Western hemispheres (Hall et al., 2009).

*Lucilia bufonivora* parasitizes mainly wild hosts (as does *W. vigil*) and its dispersal is unlikely to have been mediated by human activity. This group of taxa experienced rapid diversification that appears to have been facilitated by geographical barriers. For example, the results presented here suggest that the diversification of the most recent ancestor to *L. bufonivora* was facilitated by geographical isolation between Nearctic and Palearctic individuals, which was estimated to have occurred 3.52 mya (95% CI: 1.08–6.35 mya, Fig. 6). Certainly, in Europe it appears that the ancestral type diverged into the well-defined Palaearctic *L. bufonivora*. However, in North America it seems to have diverged into a Nearctic lineage that subsequently diversified 1 mya later into *L. elongata* and a Nearctic *L. bufonivora* (2.19 mya, 95% CI: 0.50–4.02 mya, Fig. 6). Therefore, it is suggested that *L. bufonivora* has been present in the North American continent for several million years but has remained unrecorded, possibly due to its low abundance and/or taxonomic confusion. However, there is currently insufficient reliable evidence to conclude exactly how this species migrated between continents. Nevertheless, the reciprocal monophyly between Nearctic and Palearctic parasites of amphibians suggests that this life history trait evolved before the intercontinental dispersal of the ancestral species, rather than obligate amphibian parasitism having independent origins in two different continents. The time-scaled phylogeny suggests that this dispersal occurred during the Pliocene, also a determining epoch for the intercontinental dispersal of vertebrates, including mammals, through Beringia (Cook et al., 2017). The Bering Land Bridge is also known to have mediated intercontinental dispersal of plants, amphibians, insects and parasites (Stevens et al., 2001; Contreras and Chapco, 2006; Li et al., 2015; Wen et al., 2016; Cook et al., 2017). Although there are existing reports of *L. bufonivora* from far east Asia and northern Canada (Draber-Mońko, 2013; Tantawi and Whitworth, 2014), more detailed phylogeographic studies, as well as updated surveys on the calliphorid fauna of eastern Russia and Alaska, are required to better understand the timing of the proposed divergence of Palaearctic and Nearctic *L. bufonivora*. Nevertheless, it can be concluded that *L. bufonivora* has been present in the North American continent for at least two million years but has remained unrecorded due to its relative rarity, as well as taxonomic confusion with *L. silvarum*.

### 4.3 Species composition in amphibian myiasis in North America

Using both morphological and molecular data, the present study confirmed the involvement of *L. bufonivora* in amphibian myiasis in Alberta, Canada. These reports relate to an infected western chorus frog in Pine Lake and a wood frog in Calling Lake (Table 1). It is of note that these specimens were adult flies reared from diseased amphibians and were originally labelled as ‘*L. silvarum*’ using early morphological keys (Hall, 1948). While based on only two cases, this suggests that some records of *L. silvarum* involved in amphibian myiasis in North America, particularly those identified using Hall's 1948 keys, are likely to be misidentifications. Firstly, and significantly, the keys do not include *L. bufonivora* at all, as at this time it was thought to be absent from North America. Secondly, Tantawi and Whitworth (2014) noted that there were several specimens of *L. bufonivora* mislabelled as ‘*L. silvarum*’ in Canadian insect collections (with 1954 as the earliest collection record). Therefore, the species composition of flies associated with amphibian myiasis in North America and reports of *L. silvarum* being involved in the disease remain confused and more research is required to resolve this issue. Nevertheless, further misidentifications can now be prevented by using the sequencing approach presented in this study (i.e. using *ITS2* and *COX1*) in combination with up-to-date morphological keys (Tantawi and Whitworth, 2014).

## 5 Conclusion

Within the genus *Lucilia,* obligate parasitism and host-specificity for amphibians is likely to have evolved just once around 4 mya. It is likely that this occurred after the niche displacement of a saprophagous/facultative parasite ancestor from the carrion-fly community. Consistent paraphyly of *L. bufonivora* across single-gene phylogenies and high mtDNA sequence divergence between Palearctic and Nearctic lineages suggest on-going cryptic speciation of *L. bufonivora* facilitated by geographical isolation. A time-scaled phylogeny suggests *L. bufonivora* has been evolving independently in these two regions for at least 2 mys. Thus, this species appears to have been present in North America since this time, but, due to its relative rarity, it has remained unrecorded by taxonomists until relatively recently (Tantawi and Whitworth, 2014). This is the first positive DNA-based identification of *L. bufonivora* from two confirmed cases of amphibian myiasis in North America.
